# Metamizole does not affect fracture healing in a murine ischemia model

**DOI:** 10.3389/fphys.2025.1630268

**Published:** 2025-07-09

**Authors:** Christian Schönbeck, Janine Stutz, Sebastian T. Schreiber, Lukas Keller, Chiara Siep, Wolfgang Metzger, Mario Giorgi, Irene Sartini, Tobias Fritz, Tim Pohlemann, Michael D. Menger, Emmanouil Liodakis, Matthias W. Laschke, Marcel Orth

**Affiliations:** ^1^ Department of Trauma, Hand and Reconstructive Surgery, Saarland University, Homburg, Germany; ^2^ Department of Veterinary Sciences, University of Pisa, Pisa, Italy; ^3^ Institute for Clinical and Experimental Surgery, Saarland University, PharmaScienceHub (PSH), Homburg, Germany

**Keywords:** metamizole, ischemia, fracture healing, bone turnover, angiogenesis, mouse

## Abstract

Metamizole is a commonly used analgesic drug in clinical fracture management, which does not affect the healing process under physiological conditions. However, many fracture patients suffer from co-morbidities resulting in ischemic conditions with impaired bone healing. The effect of metamizole on fracture healing under ischemic conditions has not been analyzed so far. Accordingly, in this study 44 CD-1 mice underwent ligation of the deep femoral artery to induce mild ischemia in the right hind limb. The femur was then fractured and stabilized with an intramedullary lag screw and the animals were daily treated per os with 50 mg/kg metamizole (n = 23) or vehicle (control; n = 21). Serum concentrations of the active metamizole metabolites, 4-methyl-amino-antipyrine (4-MAA) and 4-amino-antipyrine (4-AA), were determined 30, 60 and 90 min after administration. Bone healing was analyzed by biomechanical, radiological, histomorphometrical and Western blot analysis at 2 and 5 weeks postoperatively. The plasma level of 4-MAA was high at all time points, whereas 4-AA peaked at 90 min after administration. Biomechanical, radiological and histomorphometrical analyses revealed no differences between metamizole-treated and control mice, while both groups showed a delayed fracture healing. Of interest, Western blot analyses of callus tissue showed an increased expression of the pro-angiogenic factor Cyr61 and the osteoanabolic runt-related transcription factor 2 (RUNX2) as well as the osteocatabolic receptor activator of NF-κB ligand (RANKL) in metamizole-treated animals when compared to controls. Taken together, these findings indicate that the application of metamizole does not affect fracture healing under ischemic conditions. Therefore, treatment with this analgesic drug may be also recommended in fracture patients suffering from co-morbidities resulting in tissue ischemia.

## 1 Introduction

Metamizole is a commonly used non-opioid analgesic drug for the treatment of acute and chronic pain (Vilcane et al., 2023; Reist et al., 2018; White, 2005). In addition to its analgesic effect, metamizole has antipyretic and spasmolytic properties (Jasiecka et al., 2014). Metamizole itself is a pro-drug that is hydrolyzed after enteral intake to its first active metabolite 4-methyl-amino-antipyrine (4-MAA) and subsequently to 4-amino-antipyrine (4-AA) (Jasiecka et al., 2014; Domínguez-Ramírez et al., 2012). Previous studies have suggested that metamizole may target the endocannabinoid system and opioid receptors (Jasiecka et al., 2014; Hinz et al., 2007; Levy et al., 1995; Rogosch et al., 2012). Additionally, metamizole has been demonstrated to inhibit cyclooxygenase (COX)-1, COX-2 and COX-3 (Hinz et al., 2007; Campos et al., 1999; Pierre et al., 2007; Chandrasekharan et al., 2002). Of those, COX-2 is essential for the process of bone healing (Gerstenfeld et al., 2003; Simon et al., 2002). However, the precise mechanism of action still remains to be fully understood (Hinz et al., 2007).

Ischemia such as peripheral arterial disease is a common co-morbidity of patients suffering from fractures (Ungprasert et al., 2018). The process of bone healing requires a highly orchestrated sequence of events to gain full osseous healing and may be negatively influenced by ischemia caused by vascular injuries or vascular diseases (Marsell and Einhorn, 2011; Dickson et al., 1995). *In vitro*, metamizole has been shown to have a detrimental effect on osteoblast-like cells (De Luna-Bertos et al., 2015). However, no inhibitory effect of metamizole on bone healing could be observed under physiological conditions in tibial fractures of rodents (Gali et al., 2014).

To the best of our knowledge, the effect of metamizole on bone healing under ischemic conditions remains to be elucidated. For this purpose, a well-established murine fracture model under ischemic conditions was used that mimics challenging clinical healing conditions (Menger et al., 2022). Moreover, it is unclear, which dosage of metamizole should be applied in rodents to achieve similar plasma levels of its active metabolites compared to the clinical use of metamizole. The aim of this study was, therefore, to analyze the effect of a clinically relevant dosage of metamizole on bone healing and to suggest whether metamizole may be used during fracture treatment under ischemic conditions in clinical practice.

## 2 Materials and methods

### 2.1 Animals

In total, 44 CD-1 mice (26 male and 18 female mice) with a body weight of 40 ± 7 g and an age of 130 ± 5 days were used. The animals were bred at the Institute for Clinical and Experimental Surgery (Saarland University, Homburg, Germany), kept at a regular 12 h (h) light and dark cycle and had free access to tap water and standard pellet food (Altromin, Lage, Germany).

The study was conducted in accordance with the German legislation on protection of animals and the NIH Guidelines for the Care and Use of Laboratory Animals and was approved was approved by the local authorities (permission number: 35/2020; State Office for Consumer Protection, Saarbrücken, Germany).

### 2.2 Surgical procedure

**FIGURE 1:**
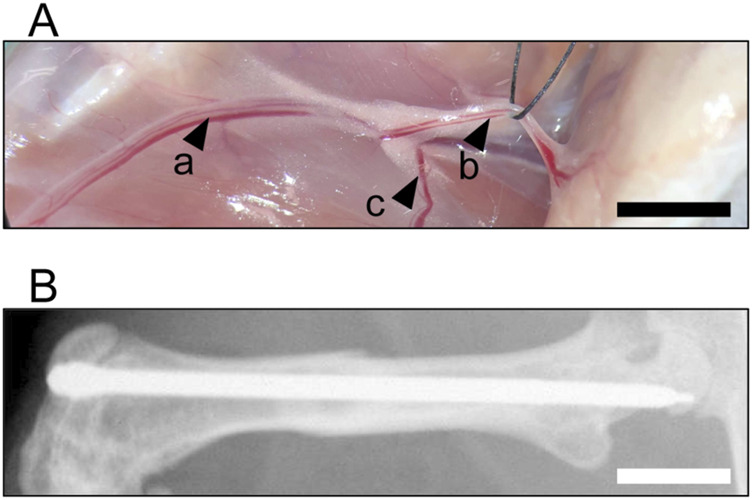
Surgical Procedure. **(A)** Identification of the femoral artery and vein (a) and epigastric artery and vein (b). Ischemia is induced by ligation of the deep femoral artery (c). **(B)** Osteosynthesis of a fractured femur after stabilization using an intramedullary lag screw at 5 weeks after surgery. Scale bar: 2 mm.

For the present study a well-established ischemic murine fracture model was used, as described previously in detail (Menger et al., 2022). For the surgical procedure, the mice were anesthetized by an intraperitoneal injection of ketamine (75 mg/kg body weight; Pharmacia, Erlangen, Germany) and xylazine (25 mg/kg body weight; Bayer, Leverkusen, Germany). Briefly, a 6-mm incision was performed medial parapatellar at the right knee in the direction of the femoral artery and vein (Figure 1A). Moderate hind limb ischemia was induced by ligation of the right deep femoral artery at the thigh. The artery was ligated using a 6–0 suture (black silk 6-0, non-absorbable; Ethicon, Raritan, United States). The knee joint capsule was then opened by incision medial to the patella, and the femoral condyles were exposed by lateralization of the patella. After drilling a hole (0.5 mm in diameter) into the intracondylar notch, an injection needle with a diameter of 0.4 mm was drilled into the intramedullary canal. Subsequently, a tungsten guidewire (0.2 mm in diameter) was inserted through the needle into the intramedullary canal. After removal of the needle, the femur was fractured by a three-point bending device. An intramedullary titanium screw (diameter: 0.5 mm; MouseScrew™, RISystem AG, Davos, Switzerland) was implanted over the guidewire to stabilize the fracture. Fracture and implant position were confirmed by radiography.

Animals were randomly assigned to one of two study groups. Animals of the metamizole group (n = 23) received 50 mg/kg metamizole (Novaminsulfon; Winthrop Arzneimittel, Mülheim-Kärlich, Germany) daily per os from the day of surgery. Animals of the control group (n = 21) received an equivalent volume of the vehicle (NaCl 0.9%; Braun, Melsungen, Germany).

Animals were sacrificed by cervical dislocation after 2 weeks (n = 13 in the control group; n = 15 in the metamizole group) or 5 weeks (n = 8 in each group) postoperatively. Directly prior to sacrifice, X-rays of the operated femurs were taken to exclude secondary dislocation of the metallic implants (Figure 1B). Femurs were harvested and used for further analyses. During outcome assessments, blinding was employed for unbiased analyses.

### 2.3 Serum analysis

To detect whether metamizole concentrations in mice are comparable to those in humans, serum levels were analyzed using high-performance liquid chromatography (HPLC). For this purpose, blood samples of operated metamizole-treated animals (n = 16) were taken under general anesthesia 30 min (min), 60 min and 90 min after the last oral administration of metamizole. The analytical methodology was carried out as described previously (Domínguez-Ramírez et al., 2012; Giorgi et al., 2015; Giorgi et al., 2018). Briefly, a 15 mL polypropylene sample bottle was used for sample extraction. A volume of 0.5 mL plasma was supplemented with 100 µL internal standard solution (Sigma Aldrich, St. Louis, United States) and mixed thoroughly for 30 s by vortexing. Subsequently, 0.1 mL sodium hydroxide solution 1 N (Titrisol, Merck, Darmstadt, Germany) was added and mixed again. The sample was supplemented with 4 mL ethyl acetate:methylene chloride (3:7 v/v; both Merck). Mixing was repeated for 30 s, followed by swirling (60 oscillations/min for 10 min) and then the mixture was centrifuged for 10 min at 10°C at 10.956 × g in a centrifuge with a 5 cm rotor radius. The organic layer was removed in 3 mL and placed in another 15 mL bottle. It was then dried at 40°C under nitrogen vaporization and redissolved with 100 µL of the mobile phase. Finally, 50 µL of this solution was analysed by HPLC. For both active metabolites of metamizole, 4-MAA and 4-AA, the detection limit was 0.03 μg/mL and the limit of quantification was 0.05 μg/mL. The concentrations for curve calibration were set at 0.05–0.1–0.25–0.5–1–5 and 10 μg/mL for both substances. The range of optimal linearity (regression lines) was defined for 4-MAA in the width of y = 0.1749x - 0.0547 (*r* ^2^ = 0.9989) and for 4-AA in the range of y = 0.3291x - 0.0293 (*r* ^2^ = 0.9995). The recovery rate for 4-MAA ranged from 90.1%–95.2% and for 4-AA from 93.9%–97.1%. The intraday and interday precision (CV%) were lower than 8.1% and 6.8%, and 9.9% and 9.4% for 4-AA and 4-MAA, respectively.

### 2.4 Biomechanical analysis

For the biomechanical analysis, the fractured right and healthy left femora of both groups were resected at 2 weeks (n = 8 in the control group; n = 10 in the metamizole group) and 5 weeks (n = 8 in each group) and freed from soft tissue. After removing the implants, callus stiffness was measured with a non-destructive test using a three-point bending device (Mini-Zwick Z 2.5; Zwick, Ulm, Germany), as described previously (Orth et al., 2022). Loading was stopped individually in every case when the actual load–displacement curve deviated more than 1% from linearity. To ensure the reproducibility of the procedure, the bone was positioned with the ventral side facing downwards, the femoral head aligned to the right, and the contact stamps centered at a distance of 6 mm from each other. The bending stiffness [N/mm] was calculated from the linear elastic part of the load-displacement diagram after applying a gradually increasing bending force with 1 mm/min. The unfractured left femora were also analyzed, serving as an internal control to account for differences in bone stiffness of metamizole-treated animals when compared to controls. All values of the fractured femora are given as absolute values and in percent of the corresponding unfractured femora. Using this non-destructive approach for biomechanical analyses, the femurs could also be used for subsequent micro-computed tomography (µCT) and histological investigations, resulting in a marked reduction of required animals according to the 3R principle.

### 2.5 Radiological analysis

X-rays (MX-20 Faxitron; X-ray Corporation, Wheeling, IL, United States) of the fractured femora were performed 2 weeks (n = 8 in the control group; n = 10 in the metamizole group) and 5 weeks (n = 8 in each group) after surgery. Fracture healing was analyzed according to the classification of Goldberg, with stage 0 indicating radiological non-union, stage 1 indicating possible union and stage 2 indicating radiological union, as described previously (Goldberg et al., 1985).

Moreover, µCT of the fractured femurs was performed 2 weeks (n = 8 in the control group; n = 10 in the metamizole group) and 5 weeks (n = 8 in each group) after surgery. Scanning was performed at a spatial resolution of 9 μm with a standardized setup (Skyscan 1,176; Bruker, Billerica, United States), as described previously (Orth et al., 2022; Orth et al., 2017). To express grey values as mineral content (bone mineral density; BMD), calcium hydroxyapatite (CaHA) phantom rods with known BMD values were used for calibration. On each transversal slide the region of interest (ROI) was contoured manually defining exclusively novel bone and excluding original cortical bone. The ROI was processed with a threshold procedure (CTAnalyzer, Bruker), which allowed for differentiation between bone and soft tissue. The thresholds to distinguish between bone and soft tissue were based on visual inspection of the images, qualitative comparison with histological sections and previous studies investigating bone repair and callus tissue by µCT (Orth et al., 2022; Morgan et al., 2009; Isaksson et al., 2009). A BMD with more than 0.410 g/cm^3^, resulting in grey values of 68–255 was defined as total mineralized bone. The following µCT parameters were calculated from the callus ROI for each specimen: bone volume (BV; mm^3^), tissue volume (TV; [mm^3^]), ratio of BV/TV (%) and trabecular parameters, such as trabecular number (TbN; [1/mm]), trabecular separation (TbSp; [mm]) and trabecular thickness (TbTh [mm]).

### 2.6 Histomorphometric analysis

For histomorphometric analyses, bones were fixed in 4% phosphate-buffered formalin for 24 h and decalcified in ethylenediaminetetraacetic acid (EDTA) solution for 14 days. Dehydration was carried out in an ascending alcohol series. After embedding decalcified bones in paraffin, longitudinal sections with a thickness of 5 µm were stained with Safranin-O (at 2 weeks: n = 8 in the control group; n = 10 in the metamizole group; at 5 weeks: n = 8 in each group). At a magnification of 12.5 × (Olympus BX60 Microscope; Olympus, Shinjuku, Japan; Zeiss Axio Cam and Axio Vision 3.1, Zeiss, Jena, Germany) structural indices were calculated based on recommendations as described elsewhere (Gerstenfeld et al., 2005). For histomorphometric evaluation the following parameters were measured: (i) total periosteal callus area, (ii) bone callus area, (iii) ratio of bone tissue area/total callus area. The total periosteal callus area was defined as all osseous, cartilaginous and fibrous callus tissue outside of the cortices. Pre-existing cortical bone and endosteal callus formation were excluded. Each area was marked and calculated using the ImageJ Analysis System (NIH, Bethesda, United States).

### 2.7 Western blot

Protein expression within the callus tissue was determined by Western blot analyses, including the expression of the angiogenic markers Cyr61, CD31, the osteoclast markers osteoprotegerin (OPG) and receptor activator of NF-κB ligand (RANKL), the osteogenic marker Runt-related transcription factor 2 (RUNX2) and the proliferation marker proliferating cell nuclear antigen (PCNA). After harvesting callus tissue 2 weeks after surgery (n = 5 in each group), tissue samples were transferred in lysis buffer and stored at −80°C. After saving the whole protein fraction, proteins were separated and transferred to membranes by standard protocols and probed using anti-Cyr61 (AF4055; R&D Systems, Minneapolis, United States), anti-CD31 (77,699; Cell Signaling Technology Europe, Frankfurt, Germany), anti-OPG (MAB4591; R&D Systems), anti-RANKL (ab62516; Abcam, Cambridge, United Kingdom), anti-RUNX2 (EPR22858-106; Abcam) and anti-PCNA (HRP-60097; Proteintech, Planegg-Martinsried, Germany) antibodies. All antibodies were incubated overnight at 4°C and afterwards for 4 h at room temperature. The appropriate peroxidase-conjugated anti-IgG antibodies served as secondary antibodies (R&D Systems and Dako Agilent, California, United States). Protein expression was visualized by means of luminol-enhanced chemiluminescence after exposure of the membrane to the Intas ECL Chemocam Imager (Intas Science Imaging Instrument GmbH, Göttingen, Germany). To correct for unequal loading, signals were normalized to β-actin signals (Santa Cruz Biotechnology, Heidelberg, Germany).

### 2.8 Statistics

All data are given as means ± standard error of the mean (SEM). The needed sample size was initially computed. For parametric data (Shapiro-Wilk test), the comparison between two groups was carried out using Student’s t-test after determining the equality of variance (Brown-Forsyte test), while analyses of three groups (serum analysis) were performed by one-way ANOVA, including the correction of the α-error according to Bonferroni probabilities to compensate for multiple comparisons. For non-parametric data, the comparison between two groups was carried out using the Mann-Whitney U-test, while analyses of three groups (serum analysis) were performed by one-way ANOVA on Ranks, followed by a Dunn’s test for all pairwise comparisons. The statistical analyses were performed using the SigmaPlot software 13.0 (Systat Software GmbH, Erkrath, Germany). A *p*-value <0.05 was considered to indicate significant differences.

## 3 Results

### 3.1 Serum analysis

**FIGURE 2:**
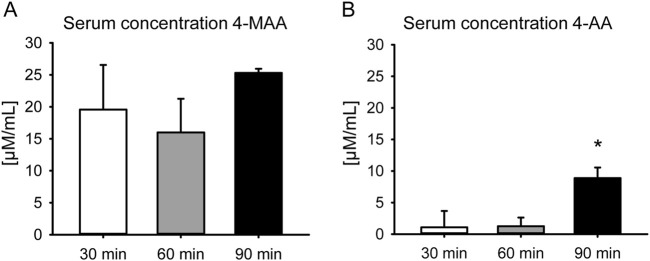
Serum concentrations of active metabolites of metamizole. **(A)** Serum concentration of 4-MAA at 30 min (white bar; n = 5), 60 min (grey bar; n = 7) and 90 min (black bar; n = 4) after administration of metamizole. **(B)** Serum concentration of 4-AA at 30 min (white bar; n = 5), 60 min (grey bar; n = 7) and 90 min (black bar; n = 4) after administration of metamizole. Mean ± SEM; *p < 0.05 vs. 60 min.

HPLC revealed high levels of 4-MAA at all investigated time points after administration of metamizole (Figure 2A). 4-AA was found to be low at 30 and 60 min and significantly higher concentrated at 90 min after administration (Figure 2B).

### 3.2 Biomechanical analysis

**FIGURE 3:**
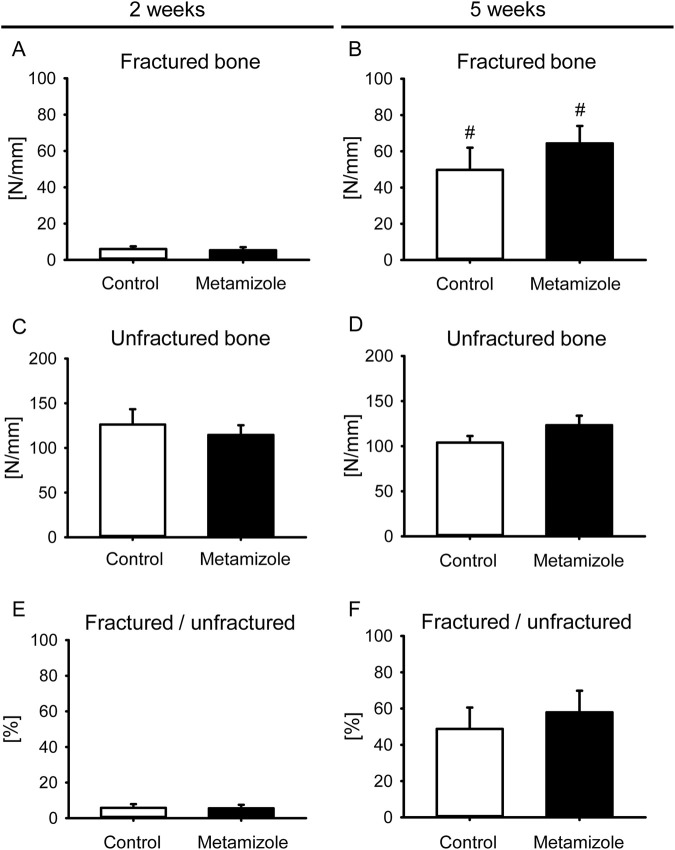
Biomechanical analysis of mouse femurs. **(A,B)** Bending stiffness of fractured control (white; n = 8) and metamizole-treated (black; n = 10/8) femurs at 2 weeks **(A)** and 5 weeks **(B)** after surgery. **(C,D)** Bending stiffness of unfractured control (white; n = 8) and metamizole-treated (black; n = 10/8) femurs at 2 weeks **(C)** and 5 weeks **(D)** after surgery. **(E,F)** Ratio of bending stiffness of fractured to unfractured control (white; n = 8) and metamizole-treated (black; n = 10/8) femurs at 2 weeks **(E)** and 5 weeks **(F)** after surgery. Mean ± SEM. ^#^p < 0.05 vs. metamizole/control at 2 weeks.

Femurs of the metamizole and control group presented with a low bending stiffness at 2 weeks after surgery and a higher stiffness at 5 weeks after surgery (Figure 3). Intragroup comparisons revealed a significant increase of bending stiffness at 5 weeks after surgery compared to results at 2 weeks (Figures 3A,B). No significant differences could be observed between both groups for fractured femurs at 2 weeks (Figure 3A) and 5 weeks (Figure 3B) and for healthy controls (Figures 3C,D). The ratio of biomechanical stiffness between fractured and unfractured bones was very low at 2 weeks and indicated an incomplete osseous stability of fractured femurs at 5 weeks after surgery in both groups (Figures 3E,F). This shows a delayed bone healing in the herein used ischemic fracture model. These results are in line with a previous study using this animal model (Menger et al., 2022).

### 3.3 Radiological analysis

**FIGURE 4:**
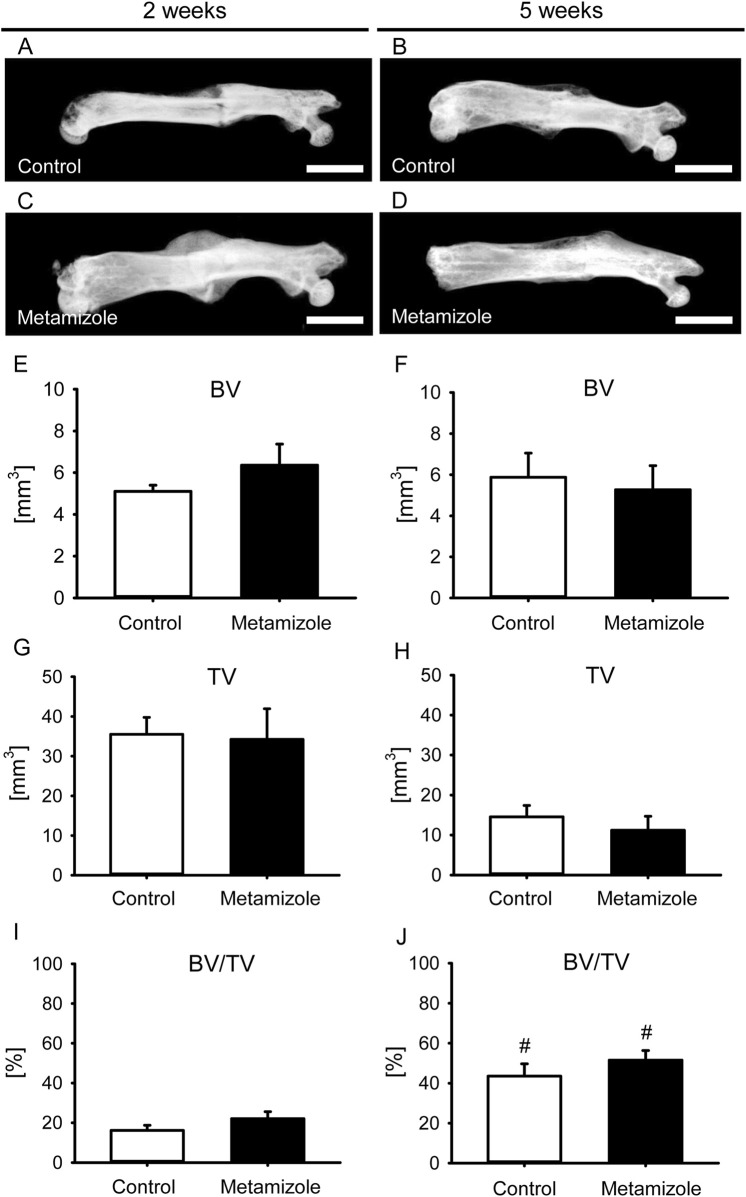
Radiological analysis of mouse femurs. **(A–D):** X-ray images of femurs at 2 weeks **(A,C)** and 5 weeks **(B,D)** after surgery of control **(A,B)** and metamizole-treated **(C,D)** animals. Scale bars: 2 mm. **(E,F)** Bone volume (BV) at 2 weeks **(E)** and 5 weeks **(F)** after surgery within the callus of control (white; n = 8) and metamizole-treated (black; n = 10/8) femurs. **(G,H)** Tissue volume (TV) at 2 weeks **(G)** and 5 weeks **(H)** after surgery within the callus of control (white; n = 8) and metamizole-treated (black; n = 10/8) femurs. **(I,J)** Ratio of bone volume to tissue volume (BV/TV) at 2 weeks **(I)** and 5 weeks **(J)** after surgery within the callus of control (white; n = 8) and metamizole-treated (black; n = 10/8) femurs. Mean ± SEM. ^#^p < 0.05 vs metamizole/control at 2 weeks.

X-rays of animals of the control and metamizole group showed signs of ongoing healing throughout the study period (Figures 4A–D). Corresponding to the X-rays, the mean Goldberg score at 2 weeks after fracture was 0.80 ± 0.13 for metamizole-treated animals and 0.75 ± 0.16 for control animals, whereas at 5 weeks the mean score for metamizole-treated animals was 1.88 ± 0.12 and for control animals 2.0 ± 0.0.

µCT analyses showed no significant differences for BV and TV at both observation time points between the two groups (Figures 4E–H). Accordingly, the BV/TV ratio did also not differ between metamizole-treated and control mice (Figures 4I,J). However, the ratio showed a significant increase of bone tissue as a fraction of TV between week 2 and 5 after surgery for each group as an indicator for ongoing bone healing. µCT analysis of the trabecular structures exhibited no significant differences between both study groups for TbN (2 weeks: control: 1.91 ± 0.33; metamizole: 2.31 ± 0.27; 5 weeks: control: 2.59 ± 0.33; metamizole: 3.47 ± 0.17), TbSp (2 weeks: control: 0.55 ± 0.05; metamizole: 0.49 ± 0.06; 5 weeks: control: 0.25 ± 0.02; metamizole: 0.22 ± 0.01) and TbTh (2 weeks: control: 0.09 ± 0.01; metamizole: 0.10 ± 0.01; 5 weeks: control: 0.17 ± 0.02; metamizole: 0.15 ± 0.02).

### 3.4 Histomorphometric analysis

**FIGURE 5:**
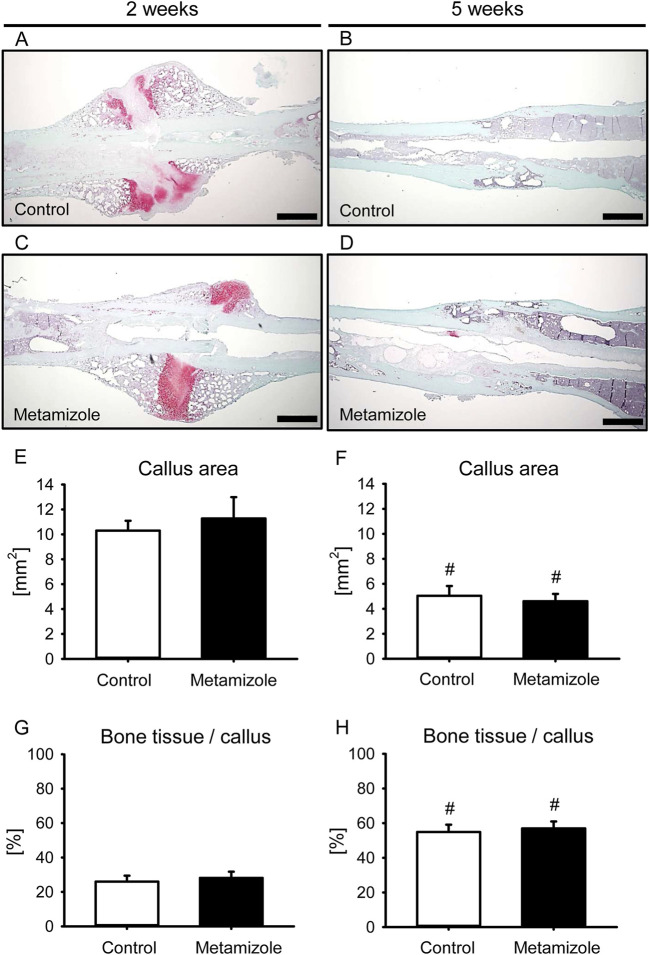
Histomorphometric analysis of mouse femurs. **(A–D):** Representative histological images of Safranin-O-stained femurs at 2 weeks **(A,C)** and 5 weeks **(B,D)** after surgery of control **(A,B)** and metamizole-treated **(C,D)** animals. Scale bars: 500 µm. **(E,F)** Total periosteal callus area of control (white; n = 8) and metamizole-treated (black; n = 10/8) femurs at 2 weeks **(E)** and 5 weeks **(F)** after surgery. **(G,H)** Fraction of bone tissue of the total callus area of control (white; n = 8) and metamizole-treated (black; n = 10/8) femurs at 2 weeks **(G)** and 5 weeks **(H)** after surgery. Mean ± SEM. ^#^p < 0.05 vs. metamizole/control at 2 weeks.

The histomorphometric analysis 2 weeks after surgery demonstrated a large callus area with mostly lack of osseous bridging in both groups (Figures 5A,C). In contrast, 5 weeks after surgery bone tissue bridged the initial fracture site in both groups (Figures 5B,D). The total callus area did not differ between the two groups (Figures 5E,F). The total callus area was smaller after 5 weeks than after 2 weeks in each group as a typical sign for remodeling of bone. Accordingly, the fraction of bone tissue of the total callus area increased over time in both groups (Figures 5G,H), whereas intergroup comparisons of this parameter revealed no differences.

### 3.5 Western blot analysis

**FIGURE 6:**
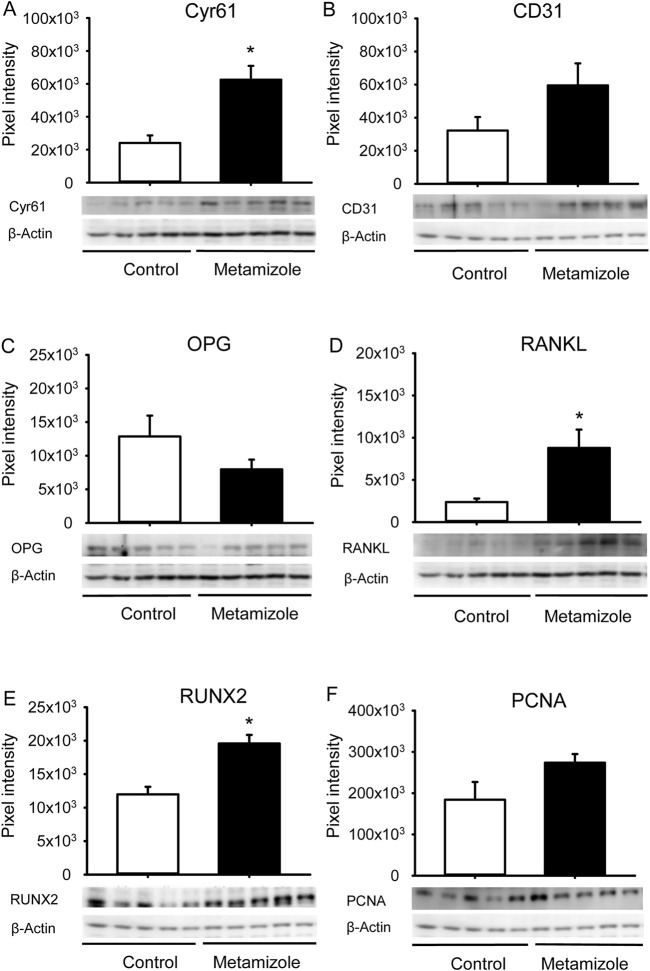
Western blot analysis of callus tissue. **(A–F):** Representative Western blots and expression of Cyr61 **(A)**, CD31 **(B)**, OPG **(C)**, RANKL **(D)**, RUNX2 **(E)**, PCNA **(F)** and β-actin **(A–F)** within the callus tissue of control (white; n = 5) and metamizole-treated (black; n = 5) femurs at 2 weeks after surgery. Mean ± SEM; ^*^p < 0.05 vs. control.

The Western blot analysis revealed a significantly higher expression of the pro-angiogenic factor Cyr61 in the callus tissue of metamizole-treated animals when compared to controls at 2 weeks after surgery (Figure 6A). The expression of CD31 did not differ between the two groups (Figure 6B). The osteoclastic marker RANKL exhibited an increased expression in the callus tissue of metamizole-treated animals, whereas expression of OPG did not show significant differences (Figures 6C,D). The expression of RUNX2 as an indicator of osteoblasts was significantly higher in the callus of metamizole-treated animals than in the callus of control animals. The expression of the proliferation marker PCNA did not differ between the two groups at this early time point (Figure 6F). These findings indicate a slightly different expression profile in metamizole-treated animals when compared to controls, indicating a trend towards a higher angiogenic activity and accelerated bone turnover at 2 weeks after surgery.

## 4 Discussion

The present study analyzed for the first time the effects of metamizole on bone healing under ischemic conditions in a well-established murine model of delayed fracture healing. For this purpose, metamizole was administered in a dosage that should mimic clinical treatment.

Metamizole is a commercially available pro-drug that is only detectable for approximately 15 min in the blood after intravenous administration (Jasiecka et al., 2014). This parent substance is not clinically effective before its conversion into the two active metabolites 4-MAA and 4-AA (Fendrich, 2000). Therefore, the serum concentrations of 4-MAA and 4-AA were assessed in the present study. While 4-MAA is an early metabolite of metamizole and a result of the gastric passage, 4-AA develops from 4-MAA during a later metabolization step after absorption (Jasiecka et al., 2014). Herein, we detected a high concentration of 4-MAA 30 min after application, which remained constant throughout the period of subsequent measurements until 90 min. This is in line with previous studies demonstrating a maximum concentration of 4-MAA 1.2–2.0 h after oral administration (Jasiecka et al., 2014). In contrast, the concentration of 4-AA was low at early time points and significantly increased at 90 min after application, which may reflect the metabolization step of 4-MAA at a later time point. Thus, these results reveal an intake and metabolization of metamizole as it would be also expected during clinical use. In patients, 4,000 mg of metamizole is considered to be the maximum daily dosage for adults and adolescents aged 15 and older (Stromer and Palladini, 2022). This daily dosage represents a common prescription for the analgesic treatment after bone injuries and corresponds to approximately 50 mg/kg body weight in an average adult of 80 kg body weight. Moreover, this dosage has been used in mice previously (Soncini et al., 2012). Accordingly, this dosage was also chosen in the present study for the treatment of mice.

Fracture healing is a well-orchestrated process in which angiogenesis and osteogenesis play pivotal roles (Cui et al., 2013). Ischemia is one of the major risk factors for complications in fracture healing (Haffner-Luntzer et al., 2019). Indeed, previous studies could show that 46% of fracture patients with vascular injuries exhibit bone healing problems and co-morbidities associated with a decreased vascularity such as diabetes, ageing and smoking influence the bone healing process due to a reduced blood flow to the fracture site (Miclau et al., 2017; Jiao et al., 2015; Hernigou and Schuind, 2013). In line with these results, it has been shown that fractures under ischemic conditions often result in non-union formation (Lu et al., 2007). The herein used animal model is characterized by a mild ischemia and has shown to induce delayed fracture healing without preventing bone regeneration (Menger et al., 2022). In the present study, intragroup comparisons between 2 and 5 weeks after surgery indicated successful osseous bridging of the fracture site. This was demonstrated in both groups by the increased biomechanical bending stiffness, the increased ratio of BV/TV in the µCT analysis and signs of remodeling histomorphometrically. However, the reduced biomechanical stability of injured femurs of approximately 50%–60% compared to healthy femurs at 5 weeks after surgery indicates that the process of bone healing is not yet completed at this late time point after surgery. Thus, the process of delayed bone healing without non-union formation by using this animal model could be confirmed in the present study.

Fractures that need to heal under pathological conditions, such as ischemia, are most likely to be negatively affected in their healing process and may even fail to heal (Haffner-Luntzer et al., 2019; Lu et al., 2007). Of interest, metamizole has been shown to increase apoptosis in osteoblast-like cells *in vitro* and to inhibit COX-1, COX-2 and COX-3, of which COX-2 is essential for bone healing (Gerstenfeld et al., 2003; Simon et al., 2002; De Luna-Bertos et al., 2015). However, under physiological conditions, metamizole did not exert a detrimental effect on bone healing in non-stabilized tibial fractures of rats (Gali et al., 2014). In line with these findings, we also did not detect any negative effects of metamizole on bone healing under ischemic conditions, as demonstrated by our biomechanical, radiological and histomorphometric results. However, under the challenging conditions of our model, metamizole slightly changed the protein expression pattern in the callus tissue at 2 weeks after surgery when compared to controls. In fact, the significantly increased expression of Cyr61, RUNX2 and RANKL in the metamizole group demonstrate an impact of metamizole and its metabolites on the angiogenic and osteo-genic mechanisms of the bone healing process under ischemic conditions. While increased expression of Cyr61 and RUNX2 indicate an angiogenic and osteoanabolic effect, the increased expression of RANKL without differences in OPG expression may indicate an increased osteocatabolic effect (Frey et al., 2012; Orth et al., 2019; Wada et al., 2006). Metamizole may, therefore, have increased the bone turnover with neither accelerating nor preventing bone healing.

In conclusion, the present study demonstrates that the application of metamizole does not affect fracture healing under challenging ischemic conditions. Although caution is naturally required, when transferring results from animal studies to applications in humans, treatment with metamizole may be also recommended for analgesia in fracture patients suffering from co-morbidities resulting in tissue ischemia.

## Data Availability

The original contributions presented in the study are included in the article/supplementary material, further inquiries can be directed to the corresponding author.
